# Ecological characterization and molecular differentiation of *Culex pipiens* complex taxa and *Culex torrentium* in eastern Austria

**DOI:** 10.1186/s13071-016-1495-4

**Published:** 2016-04-11

**Authors:** Carina Zittra, Eva Flechl, Michael Kothmayer, Simon Vitecek, Heidemarie Rossiter, Thomas Zechmeister, Hans-Peter Fuehrer

**Affiliations:** Department of Pathobiology, Institute of Parasitology, University of Veterinary Medicine Vienna, Vienna, Austria; Department of Limnology and Bio-Oceanography, University of Vienna, Vienna, Austria; Donaustraße 73, 3421 Höflein/Donau, Austria; Biological Station Lake Neusiedl, Illmitz, Burgenland Austria

**Keywords:** Mosquito, Vector, Diversity, Autecology, CQ11, Acetylcholinesterase (ACE gene)

## Abstract

**Background:**

*Culex pipiens* complex taxa differ in behaviour, ecophysiology and epidemiologic importance. Despite their epidemiologic significance, information on genetic diversity, occurrence and seasonal and spatial distribution patterns of the *Cx. pipiens* complex is still insufficient. Assessment of seasonal and spatial distribution patterns of *Culex pipiens* forms and their congener *Cx. torrentium* is crucial for the understanding of their vector–pathogen dynamics.

**Methods:**

Female mosquitoes were trapped from April–October 2014 twice a month for a 24-h time period with BG-sentinel traps at 24 sampling sites in eastern Austria, using carbon dioxide as attractant. Ecological forms of *Cx. pipiens* s.l. and their hybrids were differentiated using the CQ11 locus, and *Cx. pipiens* forms and their congener *Cx. torrentium* using the ACE-2 gene. Differential exploitation of ecological niches by *Cx. pipiens* forms and *Cx. torrentium* was analysed using likelihood ratio tests. Possible effects of environmental parameters on these taxa were tested using PERMANOVA based on distance matrices and, if significant, were modelled in nMDS ordination space to estimate non-linear relationships.

**Results:**

For this study, 1476 *Culex* spp. were sampled. *Culex pipiens* f. *pipiens* representing 87.33 % of the total catch was most abundant, followed by hybrids of both forms (5.62 %), *Cx. torrentium* (3.79 %) and *Cx. pipiens* f. *molestus* (3.25 %). Differences in proportional abundances were found between land cover classes. Ecological parameters affecting seasonal and spatial distribution of these taxa in eastern Austria are precipitation duration, air temperature, sunlight and the interaction term of precipitation amount and the Danube water level, which can be interpreted as a proxy for breeding habitat availability.

**Conclusions:**

The *Cx. pipiens* complex of eastern Austria comprises both ecologically different forms, the mainly ornithophilic form *pipiens* and the mainly mammalophilic and anthropophilic form *molestus*. Heterogeneous agricultural areas as areas of coexistence may serve as hybridization zones, resulting in potential bridge vectors between birds and humans. Occurrence, seasonal and spatial distribution patterns of the *Cx. pipiens* complex and *Cx. torrentium* and the presence of hybrids between both forms were quantified for the first time in Austria. These findings will improve the knowledge of their vector–pathogen dynamics in this country.

## Background

Species of the *Culex* (*Culex*) *pipiens* complex are hardly distinguishable due to their morphological similarity, but differ extensively in behaviour, physiology and host preference. The complex is considered to consist of seven taxa: *Cx. quinquefasciatus* Say, *Cx. pipiens pallens* Coquillet, *Cx. australicus* Dobrotworsky & Drummond, *Cx. globocoxitus* Dobrotworsky and the nominal species, *Cx. pipiens* L., comprising two genetically and ecologically distinct forms: *Culex pipiens* f. *pipiens,* which is repeatedly described and examined as ornithophilic, diapausing, anautogenous, and eurygamous, whereas *Culex pipiens* f. *molestus* is interpreted as mammophilic (and especially anthropophilic), autogenous, and stenogamous [1–4]. Both forms are known to hybridize in areas of coexistence [5, 6], potentially resulting in bridge vector populations presumed to feed mostly on birds, but also on humans [4]. However, hybridization events in central and northern Europe potentially are rare due to selective and exclusive habitat use of both forms [7–11]. Genetically distinct non-members, but often indistinguishable species from those in the *Cx. pipiens* complex are *Cx. torrentium* Martini in northern Europe, *Cx. restuans* Theob., *Cx. nigripalpus* Theob. and *Cx. salinarius* Coquillet in North America, *Cx. pervigilans* Von Bergroth in New Zealand and *Cx. vagans* Wiedemann in central and eastern Asia [12].

*Culex pipiens* complex taxa are key vectors succouring the transmission of a variety of pathogens such as avian malaria (*Plasmodium* spp.) and filarioid helminths (*Dirofilaria* spp.) [13, 14] and are considered to be main vectors of West Nile virus [15–18] and highly competent vectors for Usutu virus [19]. The presumed ornithophilic *Cx. pipiens* f. *pipiens* and *Cx. torrentium* are additionally vectors of Sindbis virus and differ in vector competence. Different transmission efficiencies for Sindbis virus (the causative agent of Ockelbo disease and Karelian fever [20, 21]) were found in *Cx. torrentium* and *Cx. pipiens* f. *pipiens* by transmission experiments in which the former species was found to be a more efficient vector [22]. Sindbis virus seroprevalence linked to migratory bird infection rates was found to fluctuate in the human population in northern Europe [23], and recently the Sindbis virus was isolated in Germany from *Cx. torrentium* [24]. Furthermore, *Cx. torrentium* is under discussion as a potentially better vector of West Nile virus than *Cx. pipiens* f. *pipiens* [25]. In addition, *Cx. pipiens* f. *molestus* was identified as vector of West Nile virus with the potential for vertical transmission within local populations [26]. Precise data on the distribution and ecology of these taxa are necessary to assess potential risks for local human populations [23] and biodiversity (e.g. [27]). The absence of stable morphological determination characters and sympatric occurrence in some *Culex* species however resulted in incomplete or erroneous data on spatial and seasonal distribution of these taxa [28]. This clearly necessitates detailed scrutinization of the ecology of *Culex* species, including *Cx. pipiens* complex taxa. However, only males or larvae of *Cx. pipiens* forms and *Cx. torrentium* can be distinguished reliably (by either the structure of the phallosome on the hypopygium [29, 30] or chaetotaxy [31]), but neither males nor larvae are of major interest in strictly epidemiological studies.

Species identification of morphologically hardly separable females of *Culex* spp. is nonetheless feasible by means of molecular methods. The mitochondrial cytochrome oxidase *c* subunit I can be used to distinguish *Cx. torrentium* from *Cx. pipiens* complex taxa [32], but attempts to identify hybrids between these taxa based on a length variation in the di-nucleotid microsatellite locus CQ11 potentially lead to some molecular misidentification of *Cx. torrentium* and *Cx. pipiens* forms [33]. Even so, utilization of two different protocols [1, 34] enables indisputable identification of *Cx. pipiens* f. *pipiens*, *Cx. pipiens* f. *molestus*, and their hybrids, *Cx. torrentium*. To our knowledge this is the first study to quantitatively examine the seasonal and spatial distribution patterns of the *Culex pipiens* forms and their sister taxon *Cx. torrentium* in Austria. This knowledge is crucial for the understanding of vector–pathogen dynamics in Austria and is furthermore essential for the implementation of appropriate mosquito surveillance and control strategies.

## Methods

### Mosquito sampling and identification

In our study 24 permanent sampling sites distributed across Lower Austria, of which two were located in Vienna (only on artificial surfaces), 14 in Lower Austria (representing predominantly artificial surfaces) and eight in Burgenland (representing predominantly agricultural areas), were monitored from April to October 2014. Mosquito communities including members of the *Culex pipiens* complex were sampled every second week for a 24-h time period using mosquito traps (Biogents®, Regensburg, Germany) baited with carbon dioxide as an attractant and were stored at −80 °C. Female mosquitoes were identified by morphological determination characters using the key of Becker et al. [31]. Identification of morphologically cryptic mosquito females of the *Cx. pipiens* complex and *Cx. torrentium* was performed following Smith & Fonseca [34] (exploiting an intron length polymorphism in the ace-2 gene to differentiate *Cx. torrentium* from *Cx. pipiens* forms) and Bahnck & Fonseca [1] (exploiting a length polymorphism of the CQ11 gene to distinguish *Cx. pipiens* f. *pipiens* and *Cx. pipiens* f. *molestus*).

### Molecular analysis

Whole genomic DNA was extracted from three legs or the head capsule of each single specimen separately using the DNeasy Blood & Tissue Kit (Qiagen, Hilden, Germany) according to the manufacturer’s protocol. To differentiate *Cx. pipiens* forms from *Cx. torrentium* partial amplification of ace-2 (*cf.* [34]) was performed using primers ACEpip, ACEpall, ACEtorr and B1246s in standard PCR protocols and cycling conditions (1 μl DNA, 5× Green Taq® Reaction Buffer, 10 pMol of each primer, 0.2 mMol of each dNTP, 1 U Taq Polymerase (Promega), ddH_2_O to 20 μl; 5 min at 95 °C, 35 × (30 s at 55 °C, 1 min at 72 °C, 30 s at 94 °C), 5 min at 72 °C). PCR products were separated using gel electrophoresis targeting 634 bp (*Cx. pipiens* forms) and 512 bp (*Cx. torrentium*) DNA fragments. Differentiation of *Cx. pipiens* f. *pipiens* and *Cx. pipiens* f. *molestus* based on partial CQ11 sequences (*cf*. [1]) was performed using primers CQ11F2, pip CQ11R and mol CQ11R in standard PCR protocols and cycling conditions (1 μl DNA, 5 × Green Taq® Reaction Buffer, 10 pMol of each primer, 2.5 mM MgCl_2_, 0.2 mM of each dNTP, 1 U Taq Polymerase (Promega), ddH_2_O to 25 μl; 5 min at 95 °C, 40 × (30 s at 54 °C, 40 s at 72 °C, 30 s at 95 °C), 5 min at 72 °C). PCR products were visualized using gel electrophoresis targeting 185 bp (*Cx. pipiens* f. *pipiens*) and 241 bp (*Cx. pipiens* f. *molestus*) DNA fragments. Further, ACE-2 and CQ11 fragments of several hybrids, *Cx. pipiens* f. *molestus*, *Cx. pipiens* f. *pipiens* were purified and directly sequenced by a commercial company (LGC Genomics, Germany) to confirm results from electrophoresis (data not shown).

Additionally, partial amplification of approximately 700 bp fragments of mitochondrial cytochrome *c* oxidase subunit I (COI) was performed using primers H15CuliCOIFw and H15CuliCOIRv (Table 1) in standard protocols and cycling conditions (1 μl DNA, 5× Green Taq® Reaction Buffer, 20 pMol of each primer, 0.2 mM of each dNTP, 1 U Taq Polymerase (Promega), ddH_2_O to 25 μl; 2 min at 95 °C, 35 × (1 min at 95 °C, 1 min at 50 °C, 1 min at 72 °C), 10 min at 72 °C) of five individuals of each taxon to ensure adequate differentiation of *Cx. torrentium* and *Cx. pipiens* forms as internal quality control (GenBank Accession numbers KU756484–KU756487).

**Table 1:** Diptera-specific PCR primers designed in this study

Specifity	Genetic marker	Primer	Sequences of primer (5′–3′)	Amplicon size
Diptera	COI	H15CuliCOIFw	AGCCATTTAATCGCGACAA	750
		H15CuliCOIRv	GGATGTCCAAAAAATCAAAATAAATGTT	

### Habitat preferences

Proportional differences in abundance of *Culex* taxa in eastern provinces of Austria (corrected against number of sampling sites per province) were assessed using a Williams-corrected likelihood ratio test (G-test) of independence [35]. Additionally, sampling sites were assigned to specific habitat types using the CORINE [Co-ordinated Information on the Environment] Land Cover [36] database to investigate potential habitat type preferences of *Culex* taxa. CLC was selected because of its high spatial resolution (1:100,000) and the methodological homogeneity used for the land cover classification. To detect differences in the abundance of *Culex* spp. in land cover classes a Williams-corrected likelihood ratio test (G-test) of goodness of fit was used.

### Seasonal and spatial variations in *Culex* spp. communities

Differential abundance of *Cx. pipiens* complex taxa per month and province was assessed using linearized graphical representations of Bray-Curtis distances produced by means of non-metric multidimensional scaling (nMDS). Meteorological data, such as temperature, air pressure, humidity, amount and duration of precipitation of 15 weather stations distributed across the sampling area were provided by the Austrian Central Institute for Meteorology and Geodynamics (ZAMG). These parameters, together with water levels of the aquatic habitats, primarily influence egg-laying, larval development and availability of larval habitats [37, 38]. Thus, 14-day means prior to the sampling date were computed to account for effects of meteorological parameters on abundance and occurrence of sampled *Culex* taxa. Effects of meteorological parameters were assessed using permutational multivariate analysis of variance (PERMANOVA) on dissimilarity matrices as implemented in the ‘vegan’ package [39], based on raw mosquito data pooled per month and province. Parameters contributing significantly to the observed patterns were subsequently modelled in ordination space using the function “ordisurf()” to estimate non-linear relationships. Species abundance in relation to communities was assessed by computing and plotting weighted average species scores. All statistical analyses were performed in the R statistical environment (R Development Core Team, 2011).

## Results

### Mosquito sampling

For this study 1476 *Culex* spp. were sampled. Cx. *pipiens* f. *pipiens* represented with 87.33 % of the total catch (*n* = 1289) the most abundant species in all provinces, followed by hybrids between *Cx. pipiens* f. *pipiens* and *Cx. pipiens* f. *molestus* (5.62 %, *n* = 83), *Cx. torrentium* (3.79 %, *n* = 56) and *Cx. pipiens* f. *molestus* (3.25 %, *n* = 48). In Vienna 244 individuals of *Cx. pipiens* f. *pipiens*, 28 *Cx. torrentium*, 25 hybrids and 13 *Cx. pipiens* f. *molestus* were collected. In Lower Austria *Cx. pipiens* f. *pipiens* was highly abundant with 612 individuals sampled, along with a further 25 hybrids, 21 *Cx. torrentium* and 17 *Cx. pipiens* f. *molestus* were collected in this province. In Burgenland 433 *Cx. pipiens* f. *pipiens*, 33 hybrids, 18 *Cx. pipiens* f. *molestus* and seven *Cx. torrentium* were identified. Sympatric occurrence of *Cx. pipiens* f. *pipiens* and *Cx. torrentium* was observed at 14 sampling locations. Sympatric occurrence of both ecological forms of *Cx. pipiens* was observed at 11 sampling localities.

### Habitat preferences

Proportional abundances of *Cx. pipiens* f. *pipiens* (G = 4.1799, X-squared df = 2, *P* = 0.1237), *Cx. pipiens* f. *molestus* (G = 0.26319, X-squared df = 2, *P* = 0.8767), hybrids (G = 0.71983, X-squared df = 2, *P* = 0.6977) and *Cx. torrentium* did not differ significantly between the provinces (G = 1.3399, X-squared df = 2, *P* = 0.5117).

Based on an analysis of differential distribution of *Culex* spp. in habitat types of CORINE Land Cover Level 1 habitat types, *Cx. pipiens* forms occur predominately in agricultural areas and *Cx. torrentium* on artificial surfaces. Significant differences in proportional abundances were found exclusively for *Cx. pipiens* f. *pipiens* in CLC level 2 habitat types (highest abundances in arable land; G = 13.312, X-squared df = 5, *P* = 0.02062) and in CLC level 3 habitat types (highest abundances in non-irrigated arable land; G = 19.766, X-squared df = 7, *P* = 0.006098). The other taxa examined did not differ significantly in their proportional abundances. Highest abundances of *Cx. pipiens* f. *pipiens* were recorded in complex cultivation patterns and non-irrigated arable land (CLC level 3), whereas *Cx. pipiens* f. *molestus* was most abundant in complex cultivation patterns (CLC level 3). Hybrids of *Cx. pipiens* f. *pipiens* and *Cx. pipiens* f. *molestus* were most abundant in non-irrigated arable land (CLC level 3) and *Cx. torrentium* in discontinuous urban fabric regions (CLC level 3).

### Seasonal and spatial variations in *Culex pipiens* complex communities

PERMANOVA indicated effects of some meteorological parameters on spatial and seasonal variations of *Culex* spp.: air temperature, sunshine duration and the amount of precipitation are possible drivers of the seasonal and spatial differentiation. Further, the interaction term of amount of precipitation and Danube water level, a proxy of breeding habitat availability, was found to structure communities (Table 2).

**Table 2:** Environmental parameters tested using PERMANOVA based on distance matrices (adonis(): ‘vegan’ package, Oksanen et al. [39])

Ecological parameter	Df	SumsOfSqs	MeanSqs	F.Model	R2	Pr(>F)
Humidity	1	0.2248	0.22478	41.529	0.06851	0.066
Precipitation duration	1	0.3366	0.33657	62.182	0.10258	0.023*
Amount of precipitation	1	0.1949	0.19489	36.007	0.05940	0.078
Air temperature	1	0.5645	0.56455	104.302	0.17207	0.007**
Sunlight duration	1	0.4238	0.42382	78.301	0.12917	0.009**
Deciduous forest cover	1	0.1795	0.17945	33.154	0.05469	0.094
Green urban areas	1	0.1080	0.10797	19.949	0.03291	0.187
Danube water level	1	0.0325	0.03252	0.6008	0.00991	0.576
Humidity:Danube water level	1	0.0839	0.08393	15.506	0.02558	0.268
Precipitation duration:Danube waterlevel	1	0.0662	0.06617	12.225	0.02017	0.318
Amount of precipitation:Danube water level	1	0.4038	0.40379	74.601	0.12307	0.020*
Air temperature:Danube water levl	1	0.1570	0.15703	29.011	0.04786	0.102
Sunshine duration:Danube water level	1	0.1186	0.11865	21.920	0.03616	0.156
Deciduous forest cover:Danube water level	1	0.1394	0.13938	25.751	0.04248	0.135
Green urban areas:Danube water level	1	0.0310	0.03097	0.5722	0.00944	0.588
Residuals	4	0.2165	0.05413		0.06599	
Total	19	32.810			100.000	

Asterisks indicate significant effects of certain environmental parameters (*, *P* ≤ 0.05; **, *P* ≤ 0.01)

**Fig. 1:**
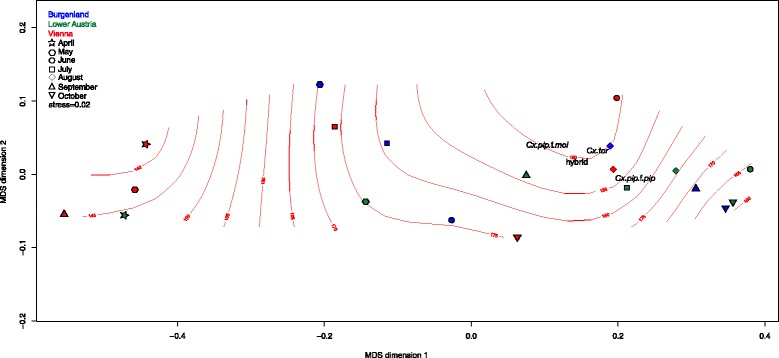
Configuration of spatial and temporal fluctuations of *Culex* spp. communities in eastern Austria in a two dimensional NMDS representation of Bray-Curtis distances. Isolines represent air temperature [°C × 10^−1^] variability throughout the sampling period modelled in ordination space using a generalized additive modelling approach as implemented in ordisurf(), deviance in ordination space explained = 44.9 %; R^2^ = <0.01

**Fig. 2:**
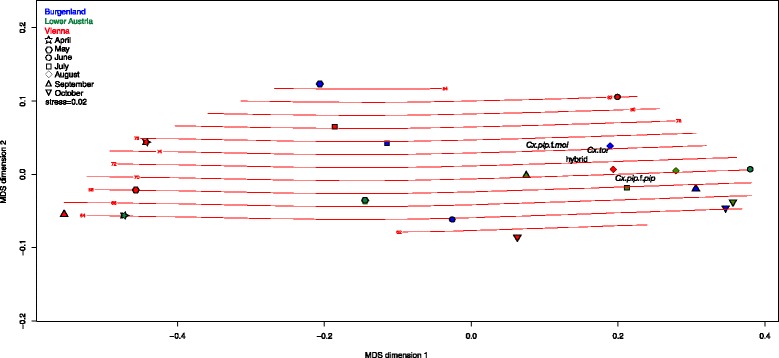
Configuration of spatial and temporal fluctuations of *Culex* spp. communities in eastern Austria in a two dimensional NMDS representation of Bray-Curtis distances. Isolines represent sunshine duration variability throughout the sampling period modelled in ordination space using a generalized additive modelling approach as implemented in ordisurf(), deviance in ordination space explained = 25.7 %; R^2^ = <0.01

*Culex pipiens* complex communities differed from one another in relation to sampling province and time. Viennese, Burgenland and Lower Austrian samples were depicted with a large spread, indicating differentiated communities throughout the sampling period (Figs. 1, 2). Viennese June and August, Lower Austrian June, July, August and September, and Burgenland August, September and October communities were loosely arranged around average weighted species scores. Communities characterized by low abundances (Viennese April, May and September, and Lower Austrian April) were more similar to one another and to all other communities sampled. May communities sampled in Burgenland and Lower Austria were relatively similar to one another and to Viennese July communities. Viennese September and Burgenland June communities were somewhat separate from the rest.

Generalized additive modelling of environmental parameters identified by PERMANOVA as potential drivers of community composition (Table 2) in ordination space suggests high abundances of all taxa to be associated with high air temperatures (Fig. 1), intermediate sunshine duration per day (Fig. 2), high duration of precipitation, and combined high Danube water levels and amounts of precipitation.

## Discussion

### Taxonomy, abundance and habitat preference of *Cx. pipiens* forms and *Cx. torrentium* in eastern Austria

Our results indicate that all taxa of the *Cx. pipiens* complex are common and widely distributed in eastern Austria. Additionally, sympatric co-occurrence of *Cx. torrentium* and *Cx. pipiens* f. *pipiens* was observed at 14 sampling localities throughout eastern Austria, predominately in discontinuous urban fabric habitats (CLC level 3). While formerly considered a rare species [40], recent surveys in Germany suggest *Cx. torrentium* to be one of the most abundant mosquitoes in Europe [41]. Previous under-representation of this species is potentially linked to cryptic taxonomic characters thwarting morphological identification (cf. [40, 41]). Distribution of adult *Cx. pipiens* f. *pipiens* and *Cx. torrentium* assessed in the present study suggests shared habitats, confirming prior studies on co-occurrence of larvae in a variety of breeding habitats (e.g. [3, 30, 41–46]). Further, the distribution of *Cx. torrentium* suggests a preference for discontinuous urban fabric, corroborating a preference for anthropogenic habitats (e.g. [25, 30, 47]).

Sympatric co-occurrence of *Cx. pipiens* forms was observed in 11 out of 24 sampling localities in Lower Austria. *Culex pipiens* forms were most abundant in agricultural areas, especially arable land and heterogeneous agricultural areas (CLC level 2), where *Cx. pipiens* f. *pipiens* and *Cx. pipiens* f. *molestus* are associated with complex cultivation patterns in co-occurrence, and *Cx. pipiens* f. *pipiens* and hybrids predominately co-occur in non-irrigated arable land (CLC level 3). Furthermore, a high proportion of hybrids in these habitat types, particularly non-irrigated arable land, characterizes them as hybridization zones. While a certain rate of hybridization of *Cx. pipiens* f. *pipiens* and *Cx. pipiens* f. *molestus* is common, these forms supposedly occur in distinct habitats types linked to their ecological peculiarities: whereas *Cx. pipiens* f. *pipiens* is reported to be ubiquitous, *Cx. pipiens* f. *molestus* is described as restricted to so-called (mostly anthropogenic) ‘underground’ habitats (e.g. in Morocco [48], Spain [7], Portugal [49] and Netherlands [5]). Also, the occurrence of *Cx. pipiens* f. *molestus* and co-occurrence of *Cx. pipiens* forms was observed in Germany and Portugal in highly urbanized areas [3, 4]. Distribution patterns of *Cx. pipiens* f. *pipiens* and *Cx. pipiens* f. *molestus* recovered in this study contrarily indicate an association of both *Cx. pipiens* f. *pipiens*, *Cx. pipiens* f. *molestus* and their hybrids with arable land and agricultural areas. This is particularly noteworthy, as an increased potential for hybridization and the formation of bridge-vectors for West Nile virus or other mosquito-borne pathogens can be expected under such circumstances. Additionally, these results clearly necessitate intensified, focused investigations on habitat preference and the distribution of *Cx. pipiens* complex taxa throughout Europe.

### Spatial and temporal variation of *Culex* taxa

Temporal variation (as pattern of Bray–Curtis distances recovered by nMDS) of *Cx. pipiens* complex taxa was observed throughout the sampling period with the most similar communities and highest abundances observed in the summer months. Meteorological parameters found to structure abundance and occurrence mostly affect developmental time and abundance (*via* larval survival and larval habitat availability) generation cycles (cf. [38, 50]). Furthermore, *Cx. pipiens* f. *pipiens* occurred earlier in the year than *Cx. torrentium* and *Cx. pipiens* f. *molestus*. This suggests somewhat higher temperature optima of these latter taxa and additionally supports the hypothesis of a more southern distribution and genesis of *Cx. pipiens* f. *molestus* [15, 51, 52] and *Cx. torrentium* [41]. However, the latter species is reported as widely absent in the Mediterranean region [41]. Putatively, this supposedly strict ornithophilic taxon is more strongly affected by other environmental parameters that also control abundance of its preferred host, e.g. habitat heterogeneity. However, the interpretability of results presented in this work is limited by the low number of specimens collected, a problem encountered by several other studies which focussed on adults (cf. [25, 53]). Nevertheless, these results indicate that a wide range of suitable larval habitats is present in eastern Austria, and, considering the low effectiveness of carbon-dioxide baited traps on certain culicid taxa, a large population of potential vectors for mosquito-borne pathogens must exist.

### Comments on efficacy of trapping methods

Interestingly, a small proportion of all collected specimens were identified as *Cx. torrentium* in our study. Under-representation of *Cx. torrentium* in carbon dioxide baited traps is commonly observed (e.g. [41, 54]), and potentially leads to an under-estimation of the proportional abundance of *Cx. torrentium*. This is particularly noteworthy, as carbon dioxide baited traps are described as attracting and catching a broad range of different mosquito species compared to other commonly used traps [55]. Differential efficacy of carbon dioxide baited traps was related to a relatively lower attractiveness of carbon dioxide to strictly ornithophilic species in comparison to anthropophilic or mammalophilic species [31, 41]. However, the supposedly mammalophilic and anthropophilic *Cx. pipiens* f. *molestus* (e.g. [31]) should consequently have been collected in higher numbers. While a large number of studies suggest strong attraction of culicidas to pure CO_2_ (e.g. [56, 57]), attractiveness of host odours seems to be controlled by prior sensitization through brief CO_2_ exposure in *Ae. aegypti* [58]. In addition, flight velocity, orientation of flight and source finding was found to increase post-CO_2_ exposure compared to naive specimens [58]. Potentially, CO_2_ acts rather as primary attractant and mediator eliciting higher sensitivity and thus the more specific response to host-odours [58]. Thus, the efficacy of carbon-dioxide-baited traps may be reduced in taxa requiring further cues to locate potent hosts. In particular, volatile substances from, for example, preen glands of birds (*cf*. [59, 60]) or serous glands of amphibia (*cf*. [61, 62]) might be more important for host localization in ornithophilic or herpetophilic taxa such as *Cx. torrentium* or *Culiseta longiareolata* [31]. Furthermore, additional host localization cues currently not considered (e.g. thermic signatures, CO_2_ concentration, optical cues) could be relevant for differential trap efficacy.

As sympatric occurrence with roughly equal abundances of *Cx. torrentium* and *Cx. pipiens* complex taxa is mainly demonstrated in larval surveys [3, 30, 46], reliable estimates of abundance of *Cx. pipiens* forms requires the analysis of larval community composition and exposition of gravid traps and ovitraps (*cf.* [41]). Putatively, such surveys will also improve data on *Cx. pipiens* f. *molestus* distribution and habitat preference as this taxon is currently potentially under-represented in the majority of studies, similar to *Cx. torrentium*.

## Conclusions

In eastern Austria both ecological forms of *Culex pipiens* exist, the mainly ornithophilic form *pipiens* and the mainly mammalophilic and anthropophilic form *molestus. Cx. pipiens* form *pipiens* is predominant in eastern Austria. Areas of co-occurrence are agricultural, peri-urban regions that may serve as areas of hybridization, resulting in bridge vectors between birds and humans. An assessment of occurrence, as well as seasonal and spatial distribution patterns of the *Cx. pipiens* forms and *Cx. torrentium* as well as the identification of hybrids was attained for the first time in Austria. These findings will contribute to the understanding of their vector–pathogen dynamics in this country.
